# Non-pathogenic *Neisseria* species of the oropharynx as a reservoir of antimicrobial resistance: a cross-sectional study

**DOI:** 10.3389/fcimb.2023.1308550

**Published:** 2023-11-22

**Authors:** Valeria Gaspari, Marielle Ezekielle Djusse, Sara Morselli, Luca Rapparini, Claudio Foschi, Simone Ambretti, Tiziana Lazzarotto, Bianca Maria Piraccini, Antonella Marangoni

**Affiliations:** ^1^ Dermatology Unit, IRCCS Azienda Ospedaliero-Universitaria di Bologna, Bologna, Italy; ^2^ Section of Microbiology, Department of Medical and Surgical Sciences, Alma Mater Studiorum - University of Bologna, Bologna, Italy; ^3^ Section of Dermatology, Department of Medical and Surgical Sciences, Alma Mater Studiorum - University of Bologna, Bologna, Italy; ^4^ Microbiology Unit, Istituto di Ricovero e Cura a Carattere Scientifico (IRCCS) Azienda Ospedaliero-Universitaria di Bologna, Bologna, Italy

**Keywords:** Neisseria, Neisseria gonorrhoeae, oropharynx, antimicrobial resistance, AMR, MSM

## Abstract

Commensal *Neisseria* species of the oropharynx represent a significant reservoir of antimicrobial resistance determinants that can be transferred to *Neisseria gonorrhoeae*. This aspect is particularly crucial in ‘men having sex with men’ (MSM), a key population in which pharyngeal co-colonization by *N. gonorrhoeae* and non-pathogenic *Neisseria* species is frequent and associated with the emergence of antimicrobial resistance. Here, we explored the antimicrobial susceptibility of a large panel of non-pathogenic *Neisseria* species isolated from the oropharynx of two populations: a group of MSM attending a ‘sexually transmitted infection’ clinic in Bologna (Italy) (n=108) and a group of males representing a ‘general population’ (n=119). We collected 246 strains, mainly belonging to *N. subflava* (60%) and *N. flavescens* (28%) species. Their antimicrobial susceptibility was evaluated assessing the minimum inhibitory concentrations (MICs) for azithromycin, ciprofloxacin, cefotaxime, and ceftriaxone using E-test strips. Overall, commensal *Neisseria* spp. showed high rates of resistance to azithromycin (90%; median MICs: 4.0 mg/L), and ciprofloxacin (58%; median MICs: 0.12 mg/L), whereas resistance to cephalosporins was far less common (<15%). *Neisseria* strains from MSM were found to have significantly higher MICs for azithromycin (p=0.0001) and ciprofloxacin (p<0.0001) compared to those from the general population. However, there was no significant difference in cephalosporin MICs between the two groups. The surveillance of the antimicrobial resistance of non-pathogenic *Neisseria* spp. could be instrumental in predicting the risk of the spread of multi-drug resistant gonorrhea. This information could be an early predictor of an excessive use of antimicrobials, paving the way to innovative screening and prevention policies.

## 1 Introduction

*Neisseria gonorrhoeae* is the causative agent of gonorrhea, one of the most common bacterial sexually transmitted infection (STI), worldwide (European Centre for Disease Prevention and Control, 2020).

This microorganism is becoming increasingly resistant to many antimicrobials, including last-resort drugs such as ceftriaxone and azithromycin, holding the status of ‘super-bug’ (Unemo et al., 2021). Antimicrobial resistance (AMR) in *N. gonorrhoeae* represents a major health concern globally, requiring elevated treatment costs and the establishment of large surveillance programs (Cole et al., 2022).

*N. gonorrhoeae* infections can be found at extra-genital sites, such as the oropharynx, mainly in ‘men who have sex with men’ (MSM) reporting unprotected oral sex (Gaspari et al., 2019).

The oropharyngeal mucosa is a particularly suitable niche for *N. gonorrhoeae* replication and persistence, and it represents a pivotal site for the emergence of multi-drug resistance (Marangoni et al., 2020a). Indeed, several studies have demonstrated that for *N. gonorrhoeae* most of resistance determinants are acquired from the commensal non-pathogenic *Neisseria* species found in the oropharyngeal environment (Marangoni et al., 2020b; Morselli et al., 2022).

It has been reported that the mosaic *penA* alleles, associated with decreased susceptibility and resistance to cephalosporins, have emerged by DNA transformation and recombination with partial *penA* genes, particularly those from commensal oropharyngeal *Neisseria* species, such as *N. perflava, N. cinerea, N. flavescens, N. polysaccharea* (Laumen et al., 2022; Manoharan-Basil et al., 2023).

Other genes responsible for the acquisition of macrolide, fluoroquinolone and/or multi-drug resistance (e.g., *mtrCDE, rplB, rplD, rplV, parC*, and *gyrA*) can be acquired by *N. gonorrhoeae* from non-pathogenic *Neisseria* species (Fiore et al., 2020; Manoharan-Basil et al., 2021; Laumen et al., 2022).

Thus, commensal *Neisseria* species have gained more and more attention, since they can act as a significant reservoir for genetic material, conferring resistance in *N*. *gonorrhoeae*, and, thus, potentially, leading to the emergence of multi-drug resistant gonorrhea.

Until now, only a few studies have assessed the antimicrobial resistance rates of commensal *Neisseria* of the oropharynx in adult populations. Among them, two surveys, carried out among MSM attending STI clinics, reported a reduced susceptibility to azithromycin and ceftriaxone in non-pathogenic *Neisseria* strains (Dong et al., 2020; Laumen et al., 2021).

Recently, Laumen et al. showed that azithromycin and ciprofloxacin resistance rates in commensal *Neisseria* are significantly higher among MSM compared to the general population, but not associated with a recent antimicrobial exposure (Laumen et al., 2022).

To expand the epidemiology of antimicrobial susceptibility in non-pathogenic *Neisseria* spp., the primary aim of this cross-sectional study was to assess the resistance patterns of oropharyngeal *Neisseria* species, by comparing two populations: (i) a group of MSM, a key population where the pharyngeal co-colonization by *N. gonorrhoeae* and non-pathogenic *Neisseria* species is particularly frequent and associated to AMR emergence (Lewis, 2013; Kenyon and Schwartz, 2018) and (ii) a group of male subjects, matched for age, ethnicity and HIV-status, representative of the general population in the same geographical area (Bologna, Italy).

To this purpose, we obtained by culture 264 pharyngeal non-pathogenic *Neisseria* strains (127 from MSM and 119 from the general population) and we assessed their susceptibility by E-test strips to four antimicrobials, namely azithromycin, ciprofloxacin, cefotaxime, and ceftriaxone.

## 2 Materials and methods

### 2.1 Study population

Two groups of subjects were considered for the study: (i) a group of white HIV-negative MSM attending the STI Outpatient Clinic of S. Orsola University Hospital of Bologna (Italy) for STI screening between January and July 2022, (ii) white males from a ‘general population’, who attended family doctors/local clinics of the Bologna area for sore throat between August and December 2022. For this last group, subjects were enrolled if negative for pharyngeal Group A *Streptococcus* (GAS; *S. pyogenes*). Information about the sexual orientation of the male subjects from the general population was not available. For both groups, individuals below the age of 18 were excluded.

(i) MSM were screened for pharyngeal sexually transmitted infections (i.e., *Chlamydia trachomatis* and *N. gonorrhoeae*) as a part of the routine clinical/diagnostic workflow. A pharyngeal swab (E-swab; Copan, Brescia, Italy) was collected from each participant and processed by Alinity m STI assay (Abbott Molecular, Illinois, USA), a real-time PCR test detecting the presence of *C. trachomatis* and/or *N. gonorrhoeae* nucleic acids. Starting from the same pharyngeal swab, a bacterial culture was performed in order to isolate commensal *Neisseria* strains (see paragraph below).

(ii) From the male individuals belonging to the ‘general population’ a pharyngeal swab (E-swab) was submitted to the Microbiology Unit of S. Orsola Hospital for the detection of GAS by bacterial culture. The same sample was used for the recovery of *Neisseria* strains as described below.

To mitigate the effect of potential confounding factors on the pharyngeal microbial communities, the two groups were matched for several variables, in addition to sex and ethnicity, such as age (no difference in the mean age) and HIV-status (all HIV-negative) (details on **Supplementary Table S1**).

The study protocol was approved by the Ethical Committee of St. Orsola-Malpighi Hospital (78/2017/U/Tess).

### 2.2 Strain collection

Pharyngeal swabs were plated onto Columbia blood agar and modified Thayer-Martin agar plates (Kima, Piove di Sacco, Italy) using the streak plate technique and incubated at 37°C at 5% CO_2_ for 48h. Afterwards, suggestive *Neisseria* colonies (oxidase-positive greyish-yellowish colonies) were isolated on chocolate agar and incubated for 24-48h at the same conditions described above. Bacterial identification at the species level was obtained through MALDI-TOF mass spectrometry (MALDI-TOF MS) (Bruker, Bremen, Germany), following manufacturer’s instructions. All the strains belonging to *Neisseria* species were collected and frozen at -80°C for further analyses. Strains belonging to *N. meningitidis* species were excluded.

### 2.3 Antimicrobial susceptibility testing

For each strain, an antimicrobial susceptibility test (AST) was performed using E-test strips (bioMérieux, Marcy-l’Étoile, France), following the ‘European Committee on Antimicrobial Susceptibility Testing’ (EUCAST) guidelines (available at https://www.eucast.org). The following antimicrobials were tested: ciprofloxacin, azithromycin, cefotaxime, and ceftriaxone. Minimum inhibitory concentration (MIC) values, expressed as mg/L, were interpreted (susceptible/resistant) using the clinical breakpoint established by EUCAST for *Neisseria gonorrhoeae* (available at https://www.eucast.org/clinical_breakpoints).

### 2.4 Data analysis and statistics

Statistical analyses were performed employing Prism 5.02 version for Windows (GraphPad Software, San Diego, CA, USA). Significant differences among the study groups (e.g., MSM vs general population; different *Neisseria* species) were searched by the Chi-square test or Mann-Whitney/Kruskal-Wallis test, based on the variables. A significance level of p < 0.05 was considered.

## 3 Results

### 3.1 Study population and strains isolated

During the study period, a total of 246 *Neisseria* strains were collected and analyzed. In particular, 127 strains were recovered from 108 MSM (89 subjects with a single strain, 19 with two strains belonging to different species) and 119 from 87 males of the general population (58 subjects with single strain, 26 with two strains, and 3 with three strains belonging to different species). In the MSM population, 9 patients suffered from pharyngeal gonorrhea (9/108; 8.3%), as indicated by the detection of *N. gonorrhoeae* by nucleic acid amplification test (NAAT).

The most frequently isolated *Neisseria* species was *N. subflava* (59.7%, 147/246), followed by *N. flavescens* (28%; 69/246). The other species were far less common: 3.2% *N. perflava* (8/246), 3.2% *N. macacae* (8/246), 2.0% *N. mucosa* (5/246), 1.6% *Neisseria* spp. (4/246), 1.2% *N. sicca* (3/246), 0.8% *N. lactamica* (2/246). The distribution of the different species among the two study groups was quite similar, except for *N. mucosa*, which was only detected in the general population, and *N. lactamica*, which was exclusively found in the MSM group (**Supplementary Table S2**). No strains of *N. gonorrhoeae* were recovered by culture.

### 3.2 Overall antimicrobial resistance

As shown in **Supplementary Table S3**, the overall resistance rate for azithromycin and ciprofloxacin exceeded 90% (91.1%) and 55% (57.7%), respectively. On the other hand, resistance to 3^rd^ generation cephalosporins (i.e., cefotaxime and ceftriaxone) was far less common (<15%). No significant differences were found among the different *Neisseria* species (**Table S2**). *N. macacae* exhibited the lowest resistance rate to all the antimicrobials tested. *N. mucosa* strains were characterized by the highest median MIC values for azithromycin and ciprofloxacin.

**Figure 1:**
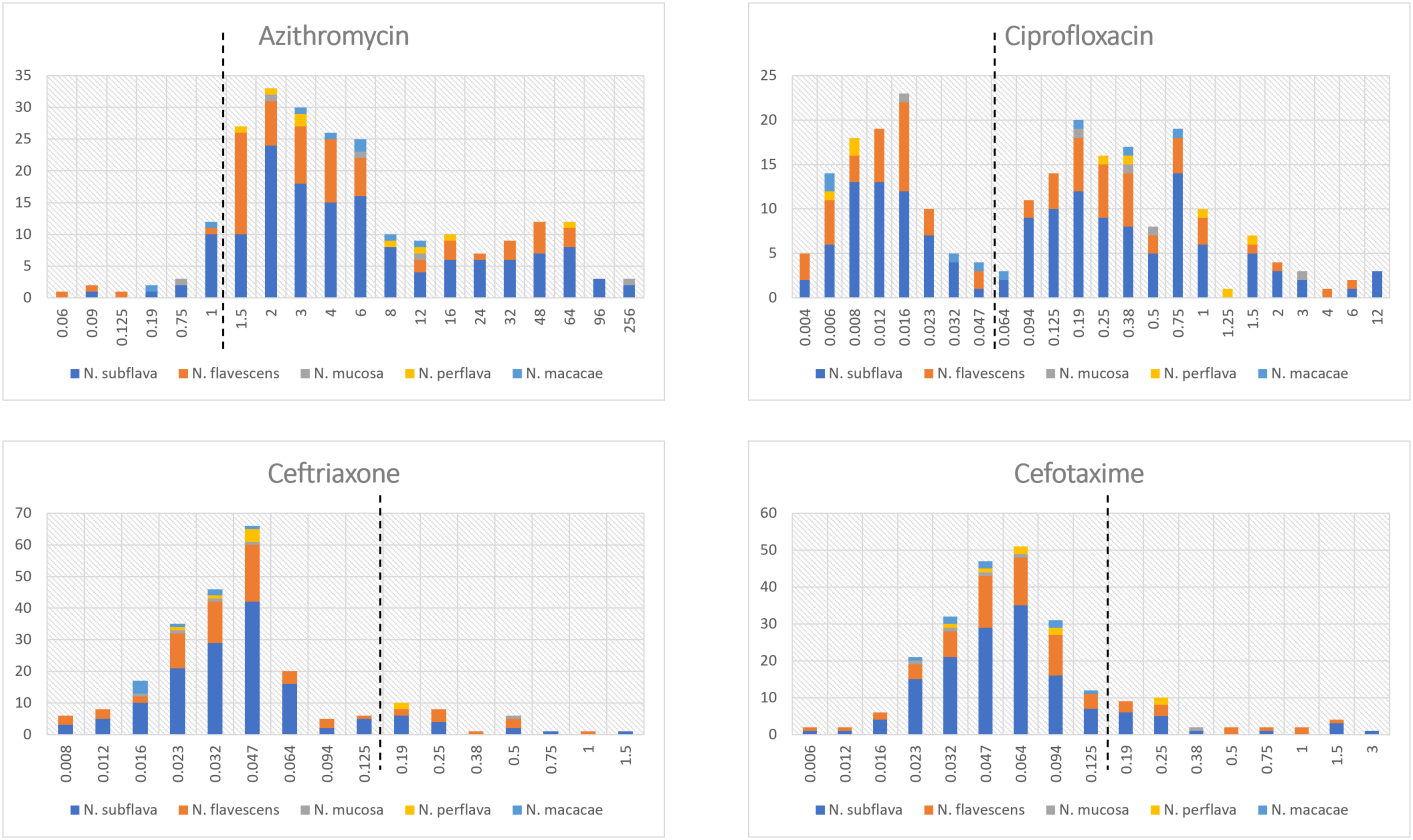
Distribution of MIC values (mg/L) stratified by *Neisseria* species. The dotted lines represent the clinical breakpoints (categorizing susceptible/resistant strains) for each antimicrobial set by the EUCAST guidelines for *Neisseria gonorrhoeae*. X-axis: MIC values; Y-axis: number of isolates.

The distribution of MIC values stratified by *Neisseria* species is displayed in **Figure 1**; **Table S4**. For azithromycin, most strains showed MIC values ranging from 1.5 to 6 mg/L (median MIC value: 4.0). In contrast, for both cefotaxime and ceftriaxone the values ranged between 0.023 and 0.064 mg/L (median MIC values: 0.06 and 0.04, respectively). Ciprofloxacin was characterized by a bimodal MIC distribution with a first peak between 0.006 and 0.016 and a second between 0.19 and 0.75 mg/L (median MIC value: 0.12).

Two strains of *Neisseria subflava* exhibited azithromycin MIC values ≥256 mg/L.

Multiple strains of different species isolated from the same subject showed similar patterns of resistance, often with identical MIC values or with a deviation of one or two-fold dilutions (data not shown).

Among the strains resistant to ceftriaxone (n=29), 89.6% of cases (26/29) also displayed resistance to azithromycin, 68.9% (20/29) to ciprofloxacin and 75.8% (22/29) to cefotaxime, thus indicating the presence of multi-drug resistant strains.

### 3.3 Comparison between the two populations

**Figure 2:**
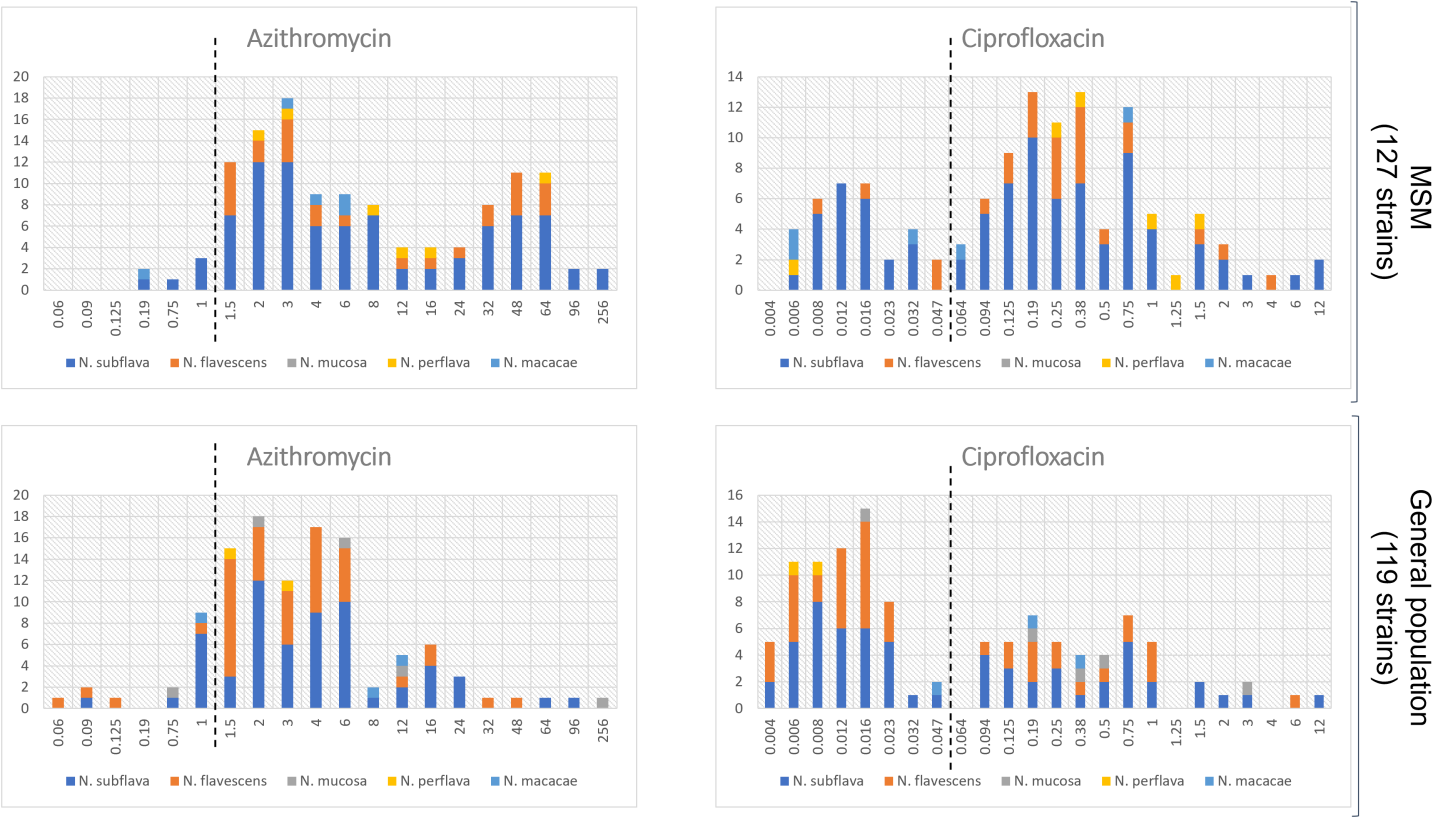
Distribution of MIC values (mg/L) for azithromycin and ciprofloxacin stratified by the two populations: MSM vs general populations. Only *Neisseria* species with at least 5 strains were considered. The dotted lines represent the clinical breakpoints (categorizing susceptible/resistant strains) for each antimicrobial set by the EUCAST guidelines for *Neisseria gonorrhoeae*. X-axis, MIC values; Y-axis, number of isolates.

**Figure 3:**
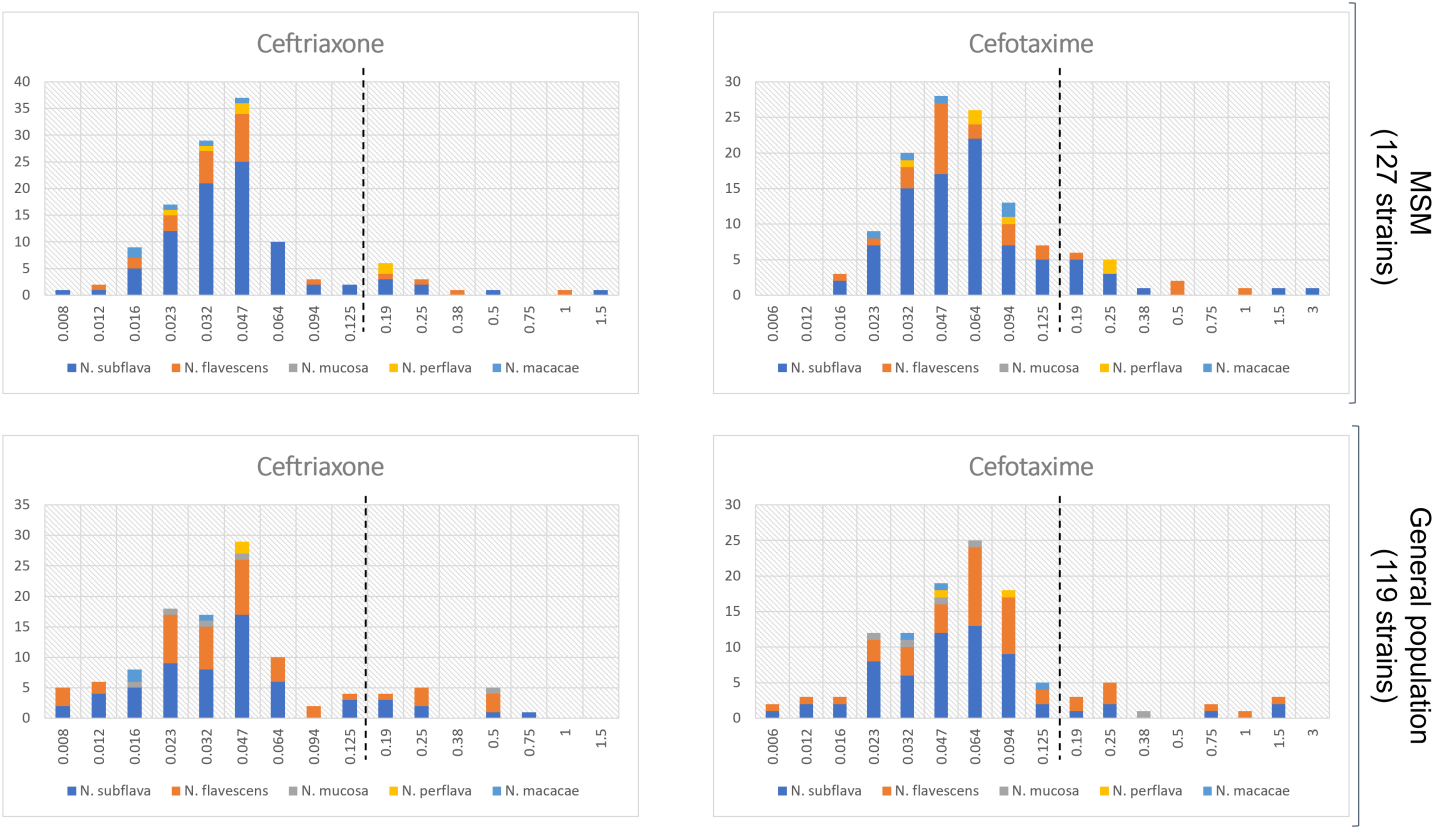
Distribution of MIC values (mg/L) for cefotaxime and ceftriaxone stratified by the two populations: MSM vs general populations. Only *Neisseria* species with at least 5 strains were considered. The dotted lines represent the clinical breakpoints (categorizing susceptible/resistant strains) for each antimicrobial set by the EUCAST guidelines for *Neisseria gonorrhoeae*. X-axis, MIC values; Y-axis, number of isolates.

Interesting data emerged when comparing resistance rates/MIC values between the two populations. As shown in **Table 1**, median MIC values for azithromycin and ciprofloxacin were significantly higher in the MSM group in comparison to the general population (p=0.0001 and p<0.0001, respectively). On the other hand, no differences were observed in the MIC values for both the cephalosporins tested (i.e., cefotaxime, p=0.78; ceftriaxone, p=0.89). Similar results were obtained when MIC values were categorized in sensitive/resistant following EUCAST *N. gonorrhoeae* clinical breakpoints (**Table S5 in the Supplementary Material**). The distribution of MIC values stratified by the two populations (MSM vs general population) is displayed in **Figure 2** (azithromycin and ciprofloxacin) and in **Figure 3** (3^rd^ generation cephalosporins).

**Table 1:** Comparison of MIC values (mg/L) of *Neisseria* strains belonging to the two populations (MSM vs general population).

MIC values	MSM (=127 strains)	General population (=119 strains)	p value
**AZI**	6.0 (2.0-32.0)	3.0 (1.5-6.0)	0.0001
**CIP**	0.19 (0.03-0.5)	0.02 (0.01-0.25)	<0.0001
**CTX**	0.04 (0.03-0.09)	0.06 (0.03-0.09)	0.78
**CRO**	0.04 (0.02-0.04)	0.04 (0.02-0.06)	0.89

Significant differences were searched by Mann-Whitney test. AZI, azithromycin; CIP, ciprofloxacin; CTX, cefotaxime; CRO, ceftriaxone.

Results are expressed as median (25th-75th percentile).

Finally, in the MSM group, pharyngeal *N. gonorrhoeae* infection did not significantly affect the detection of antimicrobial resistance in commensal *Neisseria* species for any of the drugs tested (data not shown).

## 4 Discussion

In this study, we explored the antimicrobial susceptibility of a large collection of non-pathogenic *Neisseria* species (n=246), isolated from the oropharyngeal mucosa in two distinct groups of individuals: MSM attending an STI clinic and males, representative of the general population. Considering that the pharyngeal bacterial communities could be influenced by several factors, such as sex, hormonal levels, age, and chronic conditions (Proctor and Relman, 2017; Marangoni et al., 2020a; Bach et al., 2023), the two groups of males were matched for age, ethnicity, and HIV-status. Moreover, subjects were enrolled in the same geographical area, within a close time period.

The importance of this study lies in the fact that commensal *Neisseria* species of the oropharynx play a pivotal role for the emergence of multi-drug resistant gonorrhea. In fact, they represent a significant reservoir for genetic determinants of antimicrobial resistance, which can be transferred to *N*. *gonorrhoeae* (de Block et al., 2022).

At first, we observed that *N. subflava* and *N. flavescens* were the most common pharyngeal non-pathogenic *Neisseria* species isolated from our study populations, accounting for 87% of the total of strains. In our setting, other *Neisseria* species, such as *N. mucosa* (2%), N. *lactamica* (0.8%) and *N. macacae* (3.2%) were far less common.

This distribution is in line with a study of Dong et al., reporting a high prevalence of *N. subflava* and *N. flavescens* recovered from the oropharynx of a group of MSM in Vietnam in 2016-2017 (Dong et al., 2020). In a recent survey in Belgium, *N. subflava* and *N. mucosa* were the most common isolated strains, along with some minority species (i.e., *N. cinerea, N. oralis, N. elongata*) that we did not have the chance to observe in the present study (Laumen et al., 2022).

Thus, the different distribution of pharyngeal non-pathogenic *Neisseria* species could depend on the different geographical areas, different socio-demographic factors (e.g., age, sex), different habits and behaviors (e.g., antimicrobials exposure, unsafe sex, smoking).

Second, regardless of the *Neisseria* species and population considered, we found a concerning overall rate of resistance for azithromycin (about 90%; median MIC values: 4.0 mg/L) and ciprofloxacin (about 58%; median MIC values: 0.12 mg/L). Notably, for azithromycin, we detected two strains of *N. subflava* with alarmingly high-level resistance (MIC ≥256 mg/L): this phenomenon has been associated with the acquisition of an *msrD* gene, likely from oral streptococci (de Block et al., 2021).

Thus, the study of other commensal bacteria of the oropharynx (e.g., streptococci) could expand the knowledge about the dynamics between *Neisseria* species and other microbes in term of antimicrobial resistance spread.

On the contrary, we observed that most *Neisseria* strains remained susceptible to both cefotaxime (resistance rate 13%; median MIC values: 0.06 mg/L) and ceftriaxone (resistance rate 11.7%; median MIC values: 0.04 mg/L).

Similar results emerged from a recent survey in Belgium that evaluated MICs of oropharyngeal *Neisseria* spp. isolated from 96 individuals in 2019. The authors found remarkably high rates of azithromycin resistance (median MIC values: 3.0 mg/L), with higher susceptibility to ceftriaxone (median MIC values: 0.047 mg/L) (Laumen et al., 2022).

In other countries (i.e., Vietnam), the proportion of pharyngeal *Neisseria* species with reduced susceptibility to ceftriaxone reaches about the 30%, especially in participants who used at least 1 antibiotic in the 6 months prior to the enrolment (Dong et al., 2020).

A recent systematic review on the global epidemiology of antimicrobial resistance in commensal *Neisseria* species highlights an increasing trend in MICs over time for all the antimicrobials in various geographical areas (Vanbaelen et al., 2022).

The reasons behind the increasing antimicrobial resistance in commensal *Neisseria* spp. are not fully understood at this time. Presumably, the exposure to broad-spectrum antimicrobials is able to select for antimicrobial resistance in non-pathogenic *Neisseria* species, that seem to be less susceptible to eradication compared to pathogenic *Neisseria* (de Block et al., 2021). In fact, populations with high levels of antimicrobial consumption have been shown to be at high risk for the emergence of antimicrobial resistance in commensal *Neisseria* species (Laumen et al., 2022).

In Italy, both the community consumption of antibiotics and the prevalence of bacterial resistance are higher than in other European countries (Cangini et al., 2021). Consequently, a likely explanation for the high rates of antimicrobial resistance found in commensal *Neisseria* species could be the excessive and inappropriate use of antimicrobials within the general Italian population (Cangini et al., 2021). Population level antimicrobial consumption may have selected for circulating commensal *Neisseria* species with increased MIC values.

Noteworthy results emerged when the resistance rates/MIC values were stratified by the two study populations. *Neisseria* strains from the MSM group were characterized by significantly higher MIC values for azithromycin (p=0.0001) and ciprofloxacin (p<0.0001), but not for cephalosporins (p=0.8), than those from the general population.

These results, in line with the data by Laumen et al. (Laumen et al., 2022), could be explained by several reasons. MSM are often exposed to an excess of antimicrobials, including macrolide, fluoroquinolones, cephalosporins and tetracyclines (Kenyon and Schwartz, 2018). An explanation of the large drug consumption among MSM is the practice of screening for gonorrhea and chlamydia, with high rates of asymptomatic infections detected and treated (Kenyon et al., 2020; Wardley et al., 2023). In this context, it has been observed that the pharyngeal environment of MSM using pre-exposure prophylaxis (PrEP) for HIV prevention is enriched with antibiotic resistance genes (Van Dijck et al., 2023).

In addition, MSM often represent a ‘closed’ community with high frequency of interpersonal contacts - like kissing and attending the same crowded recreational events - during which transmission of commensal microorganisms of the oropharynx may occur (Aral, 2022).

In fact, it has been shown that the oral *Neisseria* communities of partners are more similar than those of unrelated individuals. Thus, commensal *Neisseria* species can be transmitted between sexual partners, presumably through intimate kissing (Van Dijck et al., 2020).

In conclusion, we found that, in our geographical area (Bologna, Italy), non-pathogenic pharyngeal *Neisseria* species showed worrisome rates of resistance to azithromycin and ciprofloxacin. Strains from MSM were significantly less susceptible to azithromycin and ciprofloxacin, but not to cephalosporins, than those from the general population.

To the best of our knowledge this is the first report on the susceptibility of pharyngeal commensal *Neisseria* species in Italy and one of the few works that compared the resistance rates in two different groups. Although our results largely confirm those of Laumen et al. (Laumen et al., 2022), we included a significantly larger panel of *Neisseria* strains, testing them for an additional antimicrobial (i.e., cefotaxime). Moreover, we included individuals from a different geographical area (Belgium *vs* Italy), comparing only male groups (*vs* males and females) matched for several variables, potentially affecting the composition of pharyngeal microbiome.

Data about the antimicrobial resistance of non-pathogenic *Neisseria* spp. in populations where pharyngeal gonorrhea is quite common (e.g., MSM) could help predict the risk of the spread of multi-drug resistant *N. gonorrhoeae* strains. Moreover, this information could be an early predictor of an excessive use or misuse of antimicrobials, paving the way to innovative screening programs and prevention policies. In this scenario, the assessment of antimicrobial susceptibility of commensal *Neisseria* species in selected populations could be added as part of routine testing in clinical microbiology laboratories.

However, this study has a number of major limitations, including, (i) the lack of information about antibiotic exposure and about behavioral factors (e.g., number of sexual partners, sexual orientation of the general population), (ii) a single center design, (iii) the absence of strains belonging to *N. gonorrhoeae* species (iv) the culture-based approach starting from a pharyngeal swab, potentially leading to miss specific or minority commensal *Neisseria* species.

Further perspectives include the use of metagenomic approaches to better profile the pharyngeal *Neisseria* species and their related resistome. In addition, we are planning to test non-pathogenic *Neisseria* strains for other antimicrobials (i.e., doxycycline), as well as to include commensal oral microbes different from *Neisseria* species (e.g., streptococci). Finally, it would be useful to explore the resistance rates of commensal *Neisseria* in groups with different levels of antibiotic exposure, to better understand the relationship between the type/dose of drug and the risk of acquisition of antimicrobial resistance.

## Data availability statement

The original contributions presented in the study are included in the article/**Supplementary Material**. Further inquiries can be directed to the corresponding author.

## Ethics statement

The studies involving humans were approved by ethical committee of St. Orsola-Malpighi Hospital (78/2017/U/Tess). The studies were conducted in accordance with the local legislation and institutional requirements. The participants provided their written informed consent to participate in this study.

## Author contributions

VG: Investigation, Methodology, Writing – original draft. MD: Data curation, Formal analysis, Writing – original draft. SM: Data curation, Formal analysis, Writing – original draft. LR: Investigation, Writing – original draft. CF: Conceptualization, Formal analysis, Supervision, Writing – original draft, Writing – review & editing. SA: Resources, Writing – original draft. TL: Resources, Writing – original draft. BP: Conceptualization, Writing – original draft. AM: Conceptualization, Supervision, Resources, Writing – original draft.
