# Supplementary material for: Non-pathogenic Neisseria species of the oropharynx as a reservoir of antimicrobial resistance: a cross-sectional study
Source: Front Cell Infect Microbiol. 2023 Nov 22;13:1308550. doi: 10.3389/fcimb.2023.1308550 (PMC10703147; doi:10.3389/fcimb.2023.1308550)
Supplement: Supplementary file 1 [file Table_1.docx]

**SUPPLEMENTARY MATERIALS**

|  | **MSM group**  **(n=108)** | **General population (n=87)** | **p value** |
| --- | --- | --- | --- |
| Males | 100% (108/108) | 100% (87/87) | - |
| Age (mean ± SD) | 31.1 ± 10.2 | 31.0 ± 9.6 | p=0.8 |
| White ethnicity | 100% (108/108) | 100% (87/87) | - |
| Negative for HIV | 100% (108/108) | 100% (87/87) | - |

**Table S1**. Characteristics of the subjects enrolled.

|  | **MSM**  **(127 strains)** | **General population**  **(119 strains)** |
| --- | --- | --- |
| *N. subflava* | 67.7% (86/127) | 51.2% (61/119) |
| *N. flavescens* | 20.5% (26/127) | 36.1% (43/119) |
| *N. mucosa* | 0.0% (0/127) | 4.2% (5/119) |
| *N. perflava* | 4.7% (6/127) | 1.6% (2/119) |
| *N. sicca* | 0.8% (1/127) | 1.6% (2/119) |
| *N. macacae* | 3.9% (5/127) | 2.5% (3/119) |
| *N. lactamica* | 1.5% (2/127) | 0.0% (0/119) |
| *N. species* | 0.8% (1/127) | 2.5% (3/119) |

**Table S2.** Distribution of non-pathogenic pharyngeal *Neisseria* species in the two populations.

| **%R** | **All strains**  **(n=246)** | ***N. subflava***  **(n=147)** | ***N. flavescens***  **(n=69)** | ***N. mucosa***  **(n=5)** | ***N. perflava***  **(n=8)** | ***N. macacae***  **(n=8)** | ***p*** |
| --- | --- | --- | --- | --- | --- | --- | --- |
| **AZI** | 91.1%  (224/246) | 90.4%  (133/147) | 94.2%  (65/69) | 80%  (4/5) | 100%  (8/8) | 75%  (6/8) | 0.2 |
| **CIP** | 57.7%  (142/246) | 59.1%  (87/147) | 53.6%  (37/69) | 80%  (4/5) | 62.5%  (5/8) | 37.5%  (3/8) | 0.5 |
| **CTX** | 13.4%  (33/246) | 11.5%  (17/147) | 17.4%  (12/69) | 20%  (1/5) | 25%  (2/8) | 0%  (0/8) | 0.4 |
| **CRO** | 11.7%  (29/246) | 9.5%  (14/147) | 15.9%  (11/69) | 20%  (1/5) | 25%  (2/8) | 0%  (0/8) | 0.3 |

**Table S3**. Resistance rate of *Neisseria* species. Resistance was defined by using EUCAST breakpoints for *N. gonorrhoeae*. Significant differences were searched by Chi-square test. Only species with at least 5 strains were considered. AZI=azithromycin; CIP=ciprofloxacin; CTX=cefotaxime; CRO=ceftriaxone

| **MIC** | **All strains**  **(n=246)** | ***N. subflava***  **(n=147)** | ***N. flavescens***  **(n=69)** | ***N. mucosa***  **(n=5)** | ***N. perflava***  **(n=8)** | ***N. macacae***  **(n=8)** | ***p*** |
| --- | --- | --- | --- | --- | --- | --- | --- |
| **AZI** | 4.0 (2.0-12.0) | 4.0 (2.0-16.0) | 3.0 (1.5-9) | 6.0 (1.3-134.0) | 5.5 (2.2-15) | 5.0 (1.5-7.5) | 0.63 |
| **CIP** | 0.12 (0.01-0.38) | 0.12 (0.01-0.5) | 0.12 (0.01-0.38) | 0.38 (0.10-1.7) | 0.31 (0.006-1.2) | 0.05 (0.01-0.33) | 0.53 |
| **CTX** | 0.06 (0.03-0.09) | 0.06 (0.03-0.09) | 0.06 (0.04-0.09) | 0.04 (0.02-0.22) | 0.08 (0.05-0.21) | 0.04 (0.03-0.09) | 0.32 |
| **CRO** | 0.04 (0.02-0.05) | 0.04 (0.02-0.06) | 0.04 (0.02-0.06) | 0.03 (0.02-0.27) | 0.04 (0.03-0.15) | 0.02 (0.01-0.03) | 0.09 |

**Table S4.** Comparison of MIC values (mg/L) of *Neisseria* strains stratified by species. Results are expressed as median (25th-75th percentile). Significant differences were searched by Kruskal-Wallis test. Only species with at least 5 strains were considered. AZI=azithromycin; CIP=ciprofloxacin; CTX=cefotaxime; CRO=ceftriaxone

| **Resistance** | **MSM**  **(=127 strains)** | **General population**  **(=119 strains)** | **p value** |
| --- | --- | --- | --- |
| **AZI** | 94.4% (120/127) | 87.3% (104/119) | 0.05 |
| **CIP** | 70.1% (89/127) | 44.5% (53/119) | <0.0001 |
| **CTX** | 13.3% (17/127) | 13.4% (16/119) | 0.9 |
| **CRO** | 10.2% (13/127) | 13.4% (16/119) | 0.4 |

**Table S5.** Resistance rate of *Neisseria* strains among the two populations (MSM vs general population). Differences were searched by Chi-square test. AZI= azithromycin; CIP= ciprofloxacin; CTX=cefotaxime; CRO=ceftriaxone
